# Knowledge, awareness, and perceptions of meningioma epidemiology among undergraduate medical students in Enugu, Nigeria: a cross-sectional mixed-methods study

**DOI:** 10.1186/s12909-026-09269-6

**Published:** 2026-04-22

**Authors:** Godswill Uzoechina, Sumia Fatima, Treasure Osajiuba, Marvellous Elochukwu, Victoria Lemeh, Emenaha Juliet, Chukwuebuka Ugwuodo, Ebubechi Nwosu, Don-Ogwudu Deborah

**Affiliations:** 1 Faculty of Clinical Sciences, University of Nigeria Teaching Hospital (UNTH), Ituku-Ozalla, Enugu, Nigeria; 2 HopeCare Cancer Research Fellow, HopeCare Initiative, Plateau, Jos, Nigeria; 3 MBBS Graduate, Rawalpindi Medical University, Rawalpindi, Punjab Pakistan; 4 Faculty of Dentistry, University of Nigeria Teaching Hospital (UNTH), Ituku-Ozalla, Enugu, Nigeria

**Keywords:** Meningioma, Medical students, Neurooncology, Medical education, Knowledge, Nigeria

## Abstract

**Background:**

Meningiomas are among the most frequent primary brain tumours; yet undergraduate medical students’ understanding of their epidemiology remains underexplored in Nigeria. This research investigated knowledge, awareness, and perceptions of meningioma epidemiology and identified associated factors among medical students.

**Methods:**

A cross-sectional study with descriptive and mixed-method components was employed among the undergraduate medical students of the University of Nigeria undergoing clinical training at the University of Nigeria Teaching Hospital (UNTH), Enugu, from January to April, 2026. A total of 427 students were selected using stratified random sampling; 332 responded to a structured self-administered questionnaire (response rate: 78.0%). Instrument assessed socio-demographics, awareness, objective knowledge (12-item knowledge score), attitudes (Likert scale), and open-ended comments. Knowledge scores were classified into good (≥ 70%), moderate (50–69%), or poor (< 50%). Data were analysed using appropriate descriptive and inferential statistical methods. Variables were summarised using descriptive analysis, and associations were tested using χ² tests, independent t-tests, and ANOVA.

**Results:**

Respondents were predominantly clinical students, with a mean age of 21.8 ± 2.4 years, and 51.5% were male. Awareness of meningioma was high (78.9%), primarily from lectures (42.8%) and textbooks (31.3%). The mean knowledge score was 5.4 ± 2.3/12, indicating overall low knowledge, with notable gaps in age distribution (38.6%). Self-rated knowledge was low (2.7 ± 1.1) but moderately correlated with objective scores (*r* = 0.38, *p* < 0.001). Only 24.5% perceived curriculum coverage as adequate, although 88.2% recognized its importance. Neurosurgical exposure was associated with higher knowledge scores (6.2 ± 2.1 vs. 4.1 ± 2.0; *p* < 0.001). Open-ended responses reflected perceived gaps in clinical exposure, teaching quality, and curriculum integration.

**Conclusion:**

Among predominantly clinical-year undergraduate medical students at the University of Nigeria, Enugu, awareness of meningioma was high, but knowledge of its epidemiology remained suboptimal. Findings suggest that improved structured clinical exposure and integration of neurooncology into the undergraduate curriculum may enhance competence within this training context.

**Supplementary Information:**

The online version contains supplementary material available at 10.1186/s12909-026-09269-6.

## Introduction

Meningiomas are among the most common primary intracranial tumours, accounting for roughly 30–38% of brain neoplasms globally and exhibiting a higher incidence with advancing age and female sex, and contribute substantially to neuro‑oncologic disease burden in low‑ and middle‑income regions (LMICs) [1, 2]. Reports from Sub-Saharan Africa indicate that meningiomas constitute a substantial proportion of adult intracranial tumours, with hospital-based studies reporting estimates ranging approximately between 30% and 42%. However, reported proportions vary across settings, largely reflecting differences in diagnostic capacity, case ascertainment, and neurosurgical service availability. In Nigeria, aggregate hospital‑based reports spanning several decades have revealed that meningiomas constitute about 27–38% of adult intracranial tumours, and overall intracranial tumour documentation has been steadily increasing with time, probably echoing enhanced diagnostic capabilities amid mounting clinical challenges [3–5].

Nonetheless, there is limited information on the extent of knowledge, awareness, and perception of meningioma epidemiology among future practitioners in Nigeria, a key competency for future clinicians in regions with rising neuro‑oncologic challenges, therefore impeding advances in early detection and referral channels [6]. Previous studies among undergraduate medical students have shown that neurooncology and cancer-related knowledge improve with clinical exposure and structured instruction, suggesting that differences in knowledge are largely influenced by curriculum design and stage of training rather than disease-specific gaps [7, 8]. While none of these studies is specific to meningioma or CNS tumours, they highlight the value of formalised teaching in raising awareness of important disease entities in the next generation of doctors.

Furthermore, global studies focusing on healthcare providers and public knowledge of central nervous system tumours reveal significant gaps in awareness of signs and symptoms and regional epidemiologic risk factors for this disease, which may lead to diagnostic delays and less-than-ideal referral patterns [9, 10]. These results imply that even among healthcare providers, there are opportunities to strengthen disease understanding, providing further rationale for a focused evaluation in undergraduate medical students. These gaps highlight potential deficiencies that may translate into future clinical practice if not addressed during undergraduate training.

While previous studies have examined general neuro-oncology or neuroscience knowledge among medical students, there remains limited evidence focusing specifically on meningioma epidemiology as a discrete domain of undergraduate medical education in Sub-Saharan Africa. Existing literature has largely assessed broad tumour awareness without isolating disease-specific epidemiological understanding or linking knowledge outcomes to levels of clinical exposure [11].

This study addresses this gap by quantifying objective knowledge of meningioma epidemiology and integrating it with structured measures of awareness, perceptions, and neurosurgical exposure among undergraduate medical students in a Nigerian tertiary training environment. By doing so, it provides a more focused assessment of how curriculum exposure and clinical training opportunities relate specifically to understanding of a single high-burden neuro-oncological entity, thereby offering actionable insight for targeted curriculum refinement in resource-limited medical education settings.

## Materials and methods

### Study design

This was a cross-sectional study with descriptive and analytical components conducted among undergraduate medical students of the University of Nigeria undergoing clinical training at the University of Nigeria Teaching Hospital (UNTH), Enugu, Nigeria. A convergent mixed-methods design was employed, integrating quantitative survey data with qualitative analysis of open-ended responses to provide complementary insights. Data were collected between January and April 2026 using a structured self-administered questionnaire adapted from previous literature [9, 10]. The study was reported in accordance with the STROBE guidelines for observational studies.

Integration of quantitative and qualitative findings was undertaken at the interpretation stage through methodological triangulation. Quantitative results on knowledge levels, awareness patterns, and statistical associations were interpreted alongside qualitative themes from open-ended responses, which provided explanatory context for observed findings. Both strands of data were compared to identify areas of convergence, particularly regarding curriculum exposure, teaching methods, and clinical experience as influencing factors on knowledge acquisition.

### Study setting

This study was conducted at the University of Nigeria Teaching Hospital (UNTH), Enugu, Nigeria, a leading tertiary institution in Southeast Nigeria. UNTH is a tertiary hospital that serves as a clinical training centre for undergraduate medical students of the University of Nigeria; it is also a medical postgraduate training facility for the University of Nigeria (UNN), with provision of clinical services in various specialties such as neurology and neurosurgery [12].

### Study population

The study population comprised undergraduate medical students in both preclinical and clinical phases of training at the University of Nigeria. However, due to the clinical setting of data collection, the sample was predominantly composed of clinical students. This distribution reflects the natural accessibility of students during hospital-based training and was accounted for in the analysis and interpretation of findings.

### Eligibility criteria

#### Inclusion criteria

Participants were eligible if they:

- Were registered undergraduate medical students of the University of Nigeria, undergoing training at the University of Nigeria Teaching Hospital (UNTH), Enugu, during the study period.
- Were in (300LVL to 600 LVL) academic level at the time of data collection.
- Provided informed consent to participate in the study.

#### Exclusion criteria

Participants were excluded if they:

Were not actively enrolled in the undergraduate medical program during the study period (e.g., students on leave of absence, suspension).

Had prior formal training or qualification in neurology, neurosurgery, or related specialties beyond the undergraduate curriculum.

Submitted questionnaires with > 20% missing responses or missing key outcome variables (knowledge items).

Returned questionnaires with multiple inconsistent or invalid responses (e.g., selecting all options indiscriminately in single-answer questions).

Students who declined participation.

These criteria were applied to ensure data quality and reduce measurement error, consistent with standard survey research practices.

### Sample size determination

The minimum sample size was calculated using the standard formula for cross-sectional studies [13]:

$$\:\mathrm{n}=\frac{{Z}^{2}\cdot\:\mathrm{p}(1\hspace{0.17em}-\hspace{0.17em}\mathrm{p})}{{d}^{2}}$$

$$\mathrm{n}\approx\:384$$

Where:

- *Z* = 1.96 (95% confidence level).
- *p* = 0.5 (assumed prevalence due to lack of prior data).
- *d* = 0.05 (margin of error).

A non-response rate of 10% was added to the calculated sample size to obtain a final sample size of *n* = 427.

### Sampling technique and data collection

A stratified random sampling method was employed. Students were stratified according to their academic level (preclinical and clinical years), and proportionate allocation was applied to each stratum. The data collection was conducted over a one-month period in 2026. Questionnaires were distributed by trained data collectors and completed voluntarily by participants. The average completion time was 3–5 min. The non-response rate (22%) may be attributed to voluntary participation, scheduling conflicts during clinical postings, and limited availability of students during data collection periods.

### Study instrument

Data were collected using a structured, self-administered questionnaire adapted from previous literature. The instrument consisted of four domains:

- Sociodemographic and Academic Characteristics:
- Awareness of Meningioma.
- Knowledge of Meningioma Epidemiology and Clinical Features.
- Perceptions and Attitudes.
- Additionally, an open-ended question explored suggested improvements to medical education regarding brain tumours (word count was 50 words max).

Content validity was established through expert review by medical educators in neurology and medical education, ensuring clarity, relevance, and alignment with curriculum objectives. Internal consistency of Likert-scale items demonstrated acceptable reliability (Cronbach’s alpha ≥ 0.70).

### Operational definitions of study constructs

Awareness, knowledge, and perceptions were defined as distinct constructs. Awareness referred to prior familiarity with meningioma and was assessed as a binary (yes/no) response. Knowledge represented an objective understanding of meningioma epidemiology and clinical features and was measured using a 12-item structured assessment, with one point awarded for each correct response. Total scores were converted into percentages and categorised as good (≥ 70%), moderate (50–69%), or poor (< 50%). Perceptions and attitudes reflected subjective evaluations of neuro-oncology education, including perceived curriculum adequacy, relevance, and exposure, and were assessed using a 5-point Likert scale. These constructs were analysed separately to allow independent assessment of cognitive knowledge versus subjective educational appraisal among participants.

These constructs were examined jointly because they represent sequential layers of cognition and perception in medical education assessment: awareness reflects exposure, knowledge reflects factual acquisition, and perceptions/attitudes reflect interpretative and educational value judgments. Together, they provide a multidimensional evaluation of learning outcomes.

### Pretesting and validation

The questionnaire was pretested among a small cohort of medical students (10% of the intended sample). The feedback from the pilot was used to refine question clarity and structure. Content validity was ensured through expert review.

### Data analysis

Data were analysed using Python 3.11 for data cleaning, transformation, and statistical analysis. Python-based tools were used due to their flexibility for integrated data cleaning, statistical testing, and reproducible workflow management within a single analytical environment. The Pandas library was used for dataset management and preprocessing, NumPy supported numerical computation, while SciPy and StatsModels were used for inferential statistical testing, including t-tests, ANOVA, and correlation analysis. Effect sizes were reported using Cohen’s d for group comparisons, and where appropriate, 95% confidence intervals were computed to accompany effect estimates to improve the interpretability of statistical findings. This workflow ensured reproducibility, flexibility in handling mixed categorical and continuous variables, and efficient execution of both descriptive and inferential analyses in a single environment.

Qualitative data were analysed using thematic analysis following Braun and Clarke’s six-step approach [14]. Open-ended responses were analysed thematically to capture perspectives not measurable through structured items. Responses were read multiple times for familiarisation. Initial codes were generated line-by-line and grouped into 26 non-repetitive codes. These codes were clustered into sub-themes and six overarching themes. Each response was assigned to its dominant theme to calculate approximate frequencies. To ensure credibility, codes and themes were reviewed by two independent researchers, and discrepancies were resolved through discussion. Representative quotes were included to illustrate each theme.

Although open-ended responses were included within a structured questionnaire, they were analysed using thematic analysis to systematically explore patterns in participants’ perspectives. This qualitative component was used to complement the quantitative findings by providing explanatory context for observed statistical associations, and integration of both datasets was conducted during interpretation through triangulation of converging themes and statistical results.

### Ethical considerations

The research protocol was designed in accordance with guidelines for biomedical researchers involving Human Subjects and the ethical standards of the Declaration of Helsinki [15]. Approval was obtained from the ethics committee of UNTH for this study (NHREC/05/01/2008B-FW00002458-1RB00002437). Participation was voluntary, and all participants provided informed consent before data collection. To maintain confidentiality, the questionnaires were anonymised, and participants were informed that they could withdraw at any time without consequences.

## Results

### Participant demographics and academic characteristics

The mean age of the participants was 21.8 ± 2.4 years (range: 18–36), 51.5% male and 48.5% female. Most students were in the clinical phase (Year 4–6: 94.6%), while Year 3 (preclinical) students were 5.4%. Only 28.0% had reported taking a formal course in neuroscience or neurosurgery. In total, 64.8% said they had been exposed to neurosurgery in some capacity, including lectures (42.2%), clinical rotations (25.6%), and ward rounds or observerships (21.1%). Only key findings are summarised in the text; detailed values are presented in accompanying tables to avoid redundancy.

Table 1 summarises the participants’ academic and demographic characteristics.

**Table 1:** Demographic and training characteristics of participants (*N* = 332)

Characteristic	Category	*n*	%
Sex	Male	171	51.5
	Female	161	48.5
Age (years)	Mean ± SD	21.8 ± 2.4	—
	Range	18–36	—
Current year of study	Year 3 (Pre-clinical)	~ 18	~ 5.4
	Year 4	~ 180	~ 54.2
	Year 5 A / 5B	~ 110	~ 33.1
	Year 6	~ 24	~ 7.2
Phase of training	Pre-clinical (Year 3)	~ 18	~ 5.4
	Clinical (Year 4–6)	~ 314	~ 94.6
Completed a formal neuroscience/neurosurgery course?	Yes	~ 93	~ 28.0
	No / Not yet	~ 239	~ 72.0
Any exposure to neurosurgery (select all that apply)	Yes (any form)	~ 215	~ 64.8
	Lectures only	~ 140	~ 42.2
	Formal clinical rotation	~ 85	~ 25.6
	Ward rounds/observership only	~ 70	~ 21.1
	No exposure	~ 117	~ 35.2

### Awareness of meningioma

78.9% of the respondents had heard of meningiomas before. The most common first sources of information were medical lectures (42.8%), textbooks (31.3%), and online resources (18.7%). Only a small proportion reported first learning about meningiomas during clinical rotations, research activities, or conferences/workshops (< 10%). Table 2 summarises the awareness of meningioma among the respondents.

**Table 2:** Awareness and sources of knowledge about meningiomas (*N* = 332)

Item	Response	*n*	%
Ever heard of meningioma?	Yes	~ 262	~ 78.9
	No	~ 70	~ 21.1
First source of information (select all that apply)	Medical lectures	~ 142	~ 42.8
	Textbooks	~ 104	~ 31.3
	Online resources	~ 62	~ 18.7
	Clinical rotations	~ 28	~ 8.4
	Research activities	~ 18	~ 5.4
	Conferences/workshops	~ 12	~ 3.6

### Objective knowledge of meningioma epidemiology and clinical features

The average score on the objective knowledge questionnaire was 5.4 ± 2.3 over 12 questions, reflecting a moderate to low level of knowledge. The highest knowledge was observed for the origin of meningiomas (86.4%) and the benign nature of the majority of tumours (71.4%). Specifically, knowledge was poor with respect to the most commonly affected age group (38.6%) and WHO grading (34.0%). The triad of clinical symptoms (headache, seizures, and focal deficits) was correctly identified by 49.7% of the participants. The mean self-assessment of knowledge was low (2.7 ± 1.1) and correlated weakly but positively with objective scores (Spearman *r* = 0.38, *p* < 0.001).

Table 3 summarises the objective acknowledgement assessment of meningioma epidemiology.

**Table 3:** Objective knowledge of meningioma epidemiology and characteristics (*N* = 332)

Knowledge Item	Correct Answer	Correct *n*	% Correct
Arise from which structure?	Meninges	~ 287	~ 86.4
Best classified as	Primary brain tumors	~ 245	~ 73.8
In adults, among the…	Most common primary brain tumors	~ 198	~ 59.6
Most commonly affected age group	Middle-aged adults (30–60 years)	~ 128	~ 38.6
More common in	Females	~ 105	~ 31.6
SSA – among the most frequent primary brain tumours	True	~ 185	~ 55.7
Known risk factor	Ionising radiation exposure	~ 265	~ 79.8
Most meningiomas are	Benign	~ 237	~ 71.4
Most common WHO grade	Grade I	~ 113	~ 34.0
Mainstay of treatment (symptomatic)	Surgery	~ 245	~ 73.8
Most appropriate imaging modality	MRI	~ 220	~ 66.3
Correct clinical presentations (all 3 correct + 2 incorrect excluded)	Headache, Seizures, Focal deficits (no fever/weight loss)	~ 165	~ 49.7

### Perceptions and attitudes toward neurooncology education

There was strong consensus that knowledge of brain tumour epidemiology is necessary and important (mean 4.6 ± 0.8; % agreement 88.2). Yet only 24.5% reported that meningiomas and brain tumour epidemiology were sufficiently addressed in their undergraduate studies (mean 2.4 ± 1.2). Most wanted more neurooncology teaching (84.6%; mean 4.5 ± 0.9). Exposure to neurosurgery was perceived to enhance understanding (mean 4.3 ± 0.9) and influence career interest in neurology or neurosurgery (mean 3.9 ± 1.1). Table 4 summarises the perception and attitudes towards meningioma and neurooncology.

**Table 4:** Perceptions and attitudes toward meningioma/neurooncology education (*N* = 332)

Statement (1 = Strongly Disagree; 5 = Strongly Agree)	Mean ± SD	% Agree (4–5)
Meningiomas & brain tumour epidemiology are adequately covered in the curriculum	≈ 2.4 ± 1.2	≈ 24–28%
Understanding brain tumour epidemiology important for medical students	≈ 4.5–4.6 ± 0.8	≈ 87–90%
Exposure to neurosurgery improves understanding of brain tumours	≈ 4.3 ± 0.9	≈ 80–84%
Knowledge of brain tumours influences interest in a neurosurgery/neurology career	≈ 3.8–4.0 ± 1.1	≈ 65–70%
I would like more teaching on neurooncology during medical school	≈ 4.4–4.6 ± 0.9	≈ 83–87%

### Associations between exposure and knowledge

Students with any form of neurosurgery exposure scored significantly higher on the objective knowledge scores (6.2 ± 2.1), compared to those without exposure (4.1 ± 2.0; t = 7.85, *p* < 0.001, Cohen’s d = 1.02). No significant differences were observed by sex (*p* = 0.62) or year of study after adjustment (ANOVA *p* = 0.14). Chi-square test demonstrated that clinical-phase students had a significantly higher chance than the pre-clinical students to have heard of meningiomas (χ² = 12.4, *p* = 0.002).

### Qualitative insights: suggestions to improve medical education

Six themes were identified from 223 open-response answers. The most frequent was enhanced clinical and practical exposure (38%), with students stressing the need for more hands-on experience in neurology and neurosurgery: “More hands-on clinical experience in neurology and neurosurgery rotations,” and “Contact with real cases, not only knowledge in the textbooks.”

Improved teaching quality and delivery (27%) highlighted simplified foundational neurophysiology and structured rotations. Curriculum development and integration (16%) suggested including brain tumours in the core curriculum and emphasising neurosurgery postings. Technology and visual learning tools (9%) encouraged simulation and 3D imaging. Research and academic engagement (6%) recommended personal research projects and pathophysiology teaching. Faculty and institutional support (5%) emphasised qualified lecturers and adequate learning resources. Table 5 summarises the qualitative analysis of the study.

**Table 5:** Summary of qualitative themes: suggestions to improve medical education on brain tumours (*N* = 223)

Rank	Theme	*n*	% of Responses	Representative Quotes
1	Enhanced Clinical & Practical Exposure	85	38%	*“More hands-on clinical experience during neurology and neurosurgery postings.” * *“Exposure to actual cases and not just textbook knowledge.”*
2	Improved Teaching Quality & Delivery	60	27%	*“Simplifying neurophysiology from the foundation would help a lot.” * *“Properly rotate students in clinical postings and teach these topics properly.”*
3	Curriculum Development & Integration	35	16%	*“Include brain tumours in the curriculum and make neurosurgery postings compulsory.” “Special consideration to emphasise it during preclinical classes.”*
4	Technology & Visual Learning Tools	20	9%	*“Integrating 3D radiological imaging with clinical symptom tracking enhances understanding.” * *“A simulation centre where students can use VR to learn about meningioma.”*
5	Research & Academic Engagement	13	6%	*“By being encouraged to carry out personal research on the subject matter.” * *“Students ought to be taught pathophysiology and encouraged to do research.”*
6	Faculty & Institutional Support	10	5%	*“The employment of capable lecturers and usage of good learning equipment.” * *“More equipment like MRI machines should be procured to help students understand brain tumors.”*

## Discussion

### Summary of key findings

This is a study assessing the knowledge, awareness, and perception of meningioma epidemiology among undergraduate medical students in Nigeria (and probably one of the very few in sub-Saharan Africa, to the best of our knowledge). While knowledge of neuro-oncology among medical students has been explored in general terms, disease-specific assessment focusing on meningioma epidemiology remains limited in Sub-Saharan African training contexts. This distinction is important because curriculum exposure and disease burden differ significantly across specialties, and generalised neuroscience assessments may obscure specific educational gaps relevant to neuro-oncology training.

The findings revealed a moderate-to low level of objective knowledge (mean score ~ 5.4 out of a total of 12 on evidence-based items) despite relatively high general awareness (78.9% of the respondents had heard of meningiomas), with the majority being exposed to the topic initially through formal medical lectures (42.6%) and textbooks (31.4%). Awareness gained from clinical rotations or research experiences was generally low (< 10%), reinforcing an overwhelming dependence on preclinical didactic materials.

The students showed a reasonable recognition of basic features, including meningeal origin (86.4% correct responses), the fact that the majority of meningiomas are benign (71.3%), ionising radiation as an important risk factor (79.8%), and surgery as the first line of symptomatic treatment (73.8%). Yet knowledge of epidemiologic specifics was substantially poorer, including for the most affected age group (38.6% correct for middle-aged adults), sex predilection (31.6% correct for females), prevailing WHO grade (34.0% correct for Grade I), and regional context in sub-Saharan Africa (55. 7% correctly considered meningiomas as the most common or one of the most common primary brain tumors). Self-perceived knowledge was similarly low (mean ≈ 2.7 on a 1–5 scale), and with a weak association with objective performance.

Perceptions were substantially positive toward the relevance of brain tumour epidemiology in the education of medical students (mean agreement 4.6/5; ≈88–90% scored ≥ 4), but only approximately one-fourth of respondents considered the subject to be sufficiently addressed in the current curriculum (mean ≈ 2.4/5). A clear majority (≈ 84–87%) of respondents wished for more comprehensive teaching of neurooncology within the preclinical curriculum, and supported the notion that exposure to neurosurgery was advantageous for learning about brain tumours (mean 4.3/5), as well as a potential catalyst for the pursuit of a career in neurosurgery or neurology (mean ≈ 3.9/5).

Among the factors predicting higher knowledge quantitatively, exposure to neurosurgery (lectures, ward rounds/observership, or formal rotations) was the most significant, with those having been exposed to neurosurgery having significantly higher scores than those without any exposure (independent samples t-test, large effect size). This correlation reflects the predominant themes of the qualitative findings for respondents, who expressed needs including: more curriculum integration, mandatory or extended clinical neurosurgery rotations, practical/exposure in theatre, case-based teaching and visual teaching methods (3D models, videos, integrated with radiology), earlier introduction of neurooncology content, and multidisciplinary sessions or workshops. The low proportion of students first encountering meningioma during clinical rotations reflects limited early clinical exposure to neurooncology cases during undergraduate training, where structured neurosurgical teaching is often restricted to later years.

These results demonstrate a clear gap between students’ recognised need for neurooncology competence and the current preclinical-heavy, theory-dominant approach to the topic in the undergraduate curriculum. The relevance of the findings is particularly important in the context of Nigeria and sub-Saharan Africa, where meningiomas account for a significant proportion of diagnosed primary intracranial tumours, and early diagnosis and referral are largely reliant on the basic awareness of frontline physicians [1, 16]. Qualitative findings complemented the quantitative results by providing contextual explanations for the observed knowledge gaps.

### Comparison with previous literature

Our findings are consistent with and add to the limited body of literature evaluating neurooncology knowledge in undergraduate medical students, showing a coherent pattern of moderate knowledge with significant gaps in epidemiology and grading, especially in sub-Saharan Africa (SSA) [17]. In high-income countries (HICs), objective knowledge of brain tumour epidemiology and WHO grading generally attains 65–80% on core items, guided by structured neurooncology curricula and routine clinical rotations [18]. Conversely, LMICs outside SSA demonstrate moderate knowledge with continued deficiencies in epidemiological and grading data, trends that are identically reflected in our cohort’s low correct response rates for age/sex predilection (38.6%) and for WHO Grade I (34.0%) [19, 20].

Within SSA, comparable data are exceedingly limited. The nearest Nigerian study, which examined epilepsy knowledge among clinical medical students, found that brain tumours were recognised as a risk factor by 87%, and the overall mean knowledge score was 71%, with marked enhancement after neurology postings [21]. Our findings confirm the well-established positive correlation between neurosurgery exposure and knowledge (large effect size) but reveal lower meningioma-specific baseline knowledge (78.9%) and objective knowledge scores (mean ≈ 5.4/12) and suggest even more pronounced curricular gaps for primary brain tumours. Reviews of regional epidemiology have established meningiomas as the most common primary intracranial tumour in SSA (commonly 30–42% of cases), particularly in adults, but hospital-based reports are sporadic and scarce in Nigeria [22–24]. This documented burden of disease is in marked contrast to our students’ poor understanding of SSA-specific epidemiology (meningiomas were only identified by 55.7% as among the commonest primary brain tumours).

Qualitatively, participants repeatedly expressed a strong need for curriculum integration, longer clinical rotations, and more visual/practical teaching, closely mirroring results from the Brain Tumour Consortium of Africa and wider SSA neurooncology capacity analyses that reveal poor training infrastructure, rudimentary tumour registries, and minimal multidisciplinary exposure across institutions [17, 25, 26]. In contrast to HIC contexts, where early clinical exposure is standard, our students’ heavy reliance on didactic lectures and textbooks (31.4%) is consistent with the theory-dominant teaching approach reported across many LMICs, reinforcing gaps in clinical presentation, risk factors, and grading [27–29].

Therefore, this study addresses a previously unreported gap as it is the first to assess the knowledge of meningioma epidemiology among medical students in SSA. Although general awareness levels on average were similar among other LMIC reports, there were specific deficiencies in grading, sex/age distribution and regional epidemiology, deficiencies which clinical exposure was clearly shown to reduce. These trends strongly highlight the necessity for context-specific curricular adaptation in resource-constrained environments.

### Interpretation of knowledge gaps

The identified deficiencies extend beyond simple recall failures and carry direct clinical implications. Less than 40% of participants accurately identified the typical middle-aged adult peak incidence of meningiomas, 34% acknowledged that the vast majority are WHO Grade I, and slightly less than 50% could correctly identify the classic symptom triad (headache, seizures, focal deficits) as opposed to nonspecific systemic symptoms. Such deficits suggest that in this environment, future physicians may overlook meningiomas in the most affected demographic, underestimate their frequently indolent behaviour, and misattribute symptoms, resulting in potential further delays in diagnosis in a population where meningiomas are already one of the leading primary brain tumours [22].

Such inadequacies are by no means random, but rather they are the predictable outcome of the current curriculum architecture. Neurooncology is limited almost exclusively to a small handful of preclinical lectures and scattered pathology sessions, without any meningioma epidemiology dedicated teaching, and minimal reinforcement during the early clinical years [27]. The result is a knowledge base that remains theoretical and fragmented, unable to connect meningeal anatomy with real-world epidemiology, grading, or presentation.

This interpretation is powerfully reinforced by students’ own voices. Across the open-ended responses, a vast majority tied their knowledge limitations to the lack of practical reinforcement. Predictably, they consistently requested earlier and more formal clinical exposure, theatre exposure, discussions based on cases, 3D visualisation aids, radiology–pathology integration, precisely what the present didactic-heavy curriculum lacks: experience-based learning [30].

### Role of neurosurgical exposure

Exposure to neurosurgery in any capacity: lectures, ward rounds/observership, or formal clinical rotations, was the strongest predictor of higher objective knowledge scores in this survey (large effect size on independent t-test). This relationship is not only statistical but also mechanistic: real-world clinical encounters enable students to anchor the abstract concepts (meningeal origin, risk factors, imaging characteristics) to real-life presentations of patients, findings at surgery, and multidisciplinary discussions, facilitating memorisation, contextualization of information, and increases in self-efficacy beyond that obtained via passive learning [31].

Students who had been exposed only to theory (didactic lectures and textbooks) performed poorly, demonstrating that the limitations of decontextualised learning are now well-established in the literature. Lacking the iterative process of bedside correlation and clinical observation, factual recall is tenuous and poorly assimilated. By contrast, even limited experience, ward rounds, and brief observerships led to identifiable improvements, indicating that modest practical reinforcement can swiftly bridge the theory–practice gap [27].

These quantitative trends are mirrored in the qualitative data, where participants repeatedly identified clinical exposure and hands-on opportunities as the most effective route to genuine comprehension and sustained interest. Therefore, the results suggest that neurosurgical exposure should not be considered a luxury, but rather a key curricular tool with the potential to convert shallow familiarity into clinically useful understanding [31].

### Strengths and innovations of the study

This study adds context-specific evidence on how undergraduate medical students in Nigeria understand meningioma epidemiology, with a focus on both factual knowledge and educational perceptions within a clinical training environment. The findings extend existing medical education literature by linking objective knowledge gaps with students’ reported learning experiences and exposure patterns.

Methodologically, the study integrates a structured quantitative assessment with systematically analysed open-ended responses, allowing alignment between measured knowledge scores and explanatory student feedback. This dual approach provides a more complete picture of both performance and perceived drivers of learning gaps.

The analytical process was standardised using a predefined scoring framework and transparent statistical procedures implemented in Python 3.11, with consistency checks across coded variables to reduce processing error. Collectively, these elements support the reliability of the dataset and strengthen the interpretability of the findings within the limits of a single-centre cross-sectional design.

### Limitations

Several limitations should be considered when interpreting the findings of this study. First, the use of self-reported measures for exposure and perceptions may be subject to recall and social desirability bias. Second, the single-centre design limits the generalisability of the findings to other medical schools in Nigeria and across Sub-Saharan Africa, where curricular structure and clinical exposure may differ.

Although the final sample size (*n* = 332) was lower than the calculated minimum (*n* = 427), it remains within an acceptable range for cross-sectional studies and was sufficient to detect statistically significant associations. However, this reduction may have limited statistical power for certain subgroup analyses and should be considered when interpreting comparative findings.

The predominance of clinical students (94.6%) may introduce selection bias and may have resulted in an overestimation of overall knowledge levels. In addition, the 22% non-response rate may introduce non-response bias. The relatively small proportion of preclinical students further limits stratified comparisons across training levels.

Finally, the inclusion of multiple-response and open-ended items enhanced the depth of data collected but may introduce variability in interpretation and categorisation despite the use of standardised coding procedures and independent thematic review.

### Recommendations

These findings highlight the need to strengthen neuro-oncology education within the undergraduate medical curriculum. Early introduction of structured neuro-oncology teaching, supported by case-based and clinically oriented learning, is recommended to improve knowledge retention and clinical relevance.

In addition, increased structured exposure to neurosurgery through clinical rotations, ward-based teaching, and supervised clinical engagement may help bridge the gap between theoretical knowledge and practical understanding.

The incorporation of visual and technology-enhanced learning tools, such as radiological imaging, simulation-based training, and 3D anatomical models, may further support comprehension of complex neuroanatomical and pathological concepts.

Furthermore, encouraging undergraduate research and academic engagement in neuroscience-related fields may enhance sustained interest and a deeper understanding of neuro-oncology.

Finally, future multi-centre and longitudinal studies are recommended to assess changes in knowledge over time and improve the generalisability of findings across different training environments.

## Conclusions

It can be concluded that while awareness of meningiomas among undergraduate medical students is relatively high, objective knowledge remains suboptimal, particularly in key epidemiological and clinical domains. Exposure to neurosurgery was also strongly correlated with knowledge, demonstrating the value of experiential learning. Furthermore, also identified significant gaps in curriculum coverage and expressed a clear need for enhanced neurooncology education. These results emphasise the necessity for structured, early and clinically integrated teaching methodologies, complemented by practical training and innovative evidence-based tools. Strengthening neurooncology training at the undergraduate level is essential to improve competence in recognising and managing brain tumours in resource-constrained settings. 

## Supplementary Information

Supplementary Material 1.

## Data Availability

The de-identified dataset generated and analysed during this study is not publicly available due to institutional privacy policies and ethical restrictions governing research involving human participants. However, the data may be made available from the corresponding author upon reasonable request, subject to approval from the University of Nigeria Teaching Hospital (UNTH) Ethics Committee.

## Abbreviations

ANOVA: Analysis of Variance
CBTRUS: Central Brain Tumor Registry of the United States
CI: Confidence Interval
CNS: Central Nervous System
d: Effect Size (Cohen’s d)
HICs: High-Income Countries
LVL: Level (Academic Level)
LMICs: Low- and Middle-Income Countries
MRI: Magnetic Resonance Imaging
n: Sample Size
NHREC: National Health Research Ethics Committee
SD: Standard Deviation
SSA: Sub-Saharan Africa
UNN: University of Nigeria
UNTH: University of Nigeria Teaching Hospital
UGMS: Undergraduate Medical Students
VR: Virtual Reality
WHO: World Health Organisation
χ²: Chi-square Test
p: Probability Value
r: Correlation Coefficient
