# Supplementary material for: Knowledge, awareness, and perceptions of meningioma epidemiology among undergraduate medical students in Enugu, Nigeria: a cross-sectional mixed-methods study
Source: BMC Med Educ. 2026 Apr 22;26:902. doi: 10.1186/s12909-026-09269-6 (PMC13235110; doi:10.1186/s12909-026-09269-6)
Supplement: Supplementary file 1 — Supplementary Material 1. [file 12909_2026_9269_MOESM1_ESM.docx]

## **Supplementary File 1: Study Questionnaire**

**Title:** Knowledge, Awareness, and Perceptions of Meningioma Epidemiology Among Undergraduate Medical Students

### **Section A: Sociodemographic and Academic Characteristics**

1. **Age (years):** _______
2. **Sex:**
   - ☐ Male
   - ☐ Female
   - ☐ Prefer not to say
3. **Current Year of Study:**
   - ☐ Year 3 (Pre-clinical)
   - ☐ Year 4
   - ☐ Year 5
   - ☐ Year 6
4. **Phase of Training:**
   - ☐ Pre-clinical
   - ☐ Clinical
5. **Have you completed a formal neuroscience/neurosurgery course?**
   - ☐ Yes
   - ☐ No
6. **Have you had any exposure to neurosurgery? (Select all that apply)**
   - ☐ Lectures
   - ☐ Clinical rotations
   - ☐ Ward rounds/observership
   - ☐ None

### **Section B: Awareness of Meningioma**

1. **Have you ever heard of meningioma?**
   - ☐ Yes
   - ☐ No
2. **Where did you first hear about meningiomas? (Select all that apply)**
   - ☐ Medical lectures
   - ☐ Textbooks
   - ☐ Online resources
   - ☐ Clinical rotations
   - ☐ Research activities
   - ☐ Conferences/workshops

**Section C: Knowledge of Meningioma Epidemiology and Clinical Features**

1. **Meningiomas arise from which structure?**
   - ☐ Meninges
   - ☐ Brain parenchyma
   - ☐ Skull bone
   - ☐ I don’t know
2. **Meningiomas are best classified as:**

- ☐ Primary brain tumours
- ☐ Metastatic tumours
- ☐ Infectious lesions
- ☐ I don’t know

1. **In adults, meningiomas are among the:**

- ☐ Most common primary brain tumours
- ☐ Rare tumours
- ☐ Secondary tumours only
- ☐ I don’t know

1. **Which age group is most commonly affected by meningiomas?**

- ☐ Children
- ☐ Young adults (18–30 years)
- ☐ Middle-aged adults (30–60 years)
- ☐ Elderly (>70 years)
- ☐ I don’t know

1. **Meningiomas are more common in:**

- ☐ Males
- ☐ Females
- ☐ No difference
- ☐ I don’t know

1. **In Sub-Saharan Africa, meningiomas are among the most frequently diagnosed primary brain tumours:**

- ☐ True
- ☐ False
- ☐ I don’t know

1. **Which of the following is a known risk factor for meningiomas?**

- ☐ Ionising radiation exposure
- ☐ Viral infection
- ☐ Smoking
- ☐ I don’t know

1. **Most meningiomas are:**

- ☐ Benign
- ☐ Malignant
- ☐ Infectious
- ☐ I don’t know

1. **Most meningiomas are classified as which WHO grade?**

- ☐ Grade I
- ☐ Grade II
- ☐ Grade III
- ☐ I don’t know

1. **Common clinical presentations of meningiomas include (Select all that apply):**

- ☐ Headache
- ☐ Seizures
- ☐ Focal neurological deficits
- ☐ Fever
- ☐ Weight loss

1. **The mainstay of treatment for symptomatic meningiomas is:**

- ☐ Surgery
- ☐ Chemotherapy
- ☐ Antibiotics
- ☐ I don’t know

1. **The most appropriate imaging modality for diagnosing meningiomas is:**

- ☐ MRI
- ☐ X-ray
- ☐ Ultrasound
- ☐ I don’t know

### **Section D: Perceptions and Attitudes**

(Scale: 1 = Strongly Disagree, 5 = Strongly Agree)

1. **How would you rate your overall knowledge of meningiomas?**

- ☐ 1 ☐ 2 ☐ 3 ☐ 4 ☐ 5

1. **Meningiomas and brain tumour epidemiology are adequately covered in the undergraduate curriculum**

- ☐ 1 ☐ 2 ☐ 3 ☐ 4 ☐ 5

1. **Understanding brain tumour epidemiology is important for medical students**

- ☐ 1 ☐ 2 ☐ 3 ☐ 4 ☐ 5

1. **Exposure to neurosurgery improves medical students’ understanding of brain tumours**

- ☐ 1 ☐ 2 ☐ 3 ☐ 4 ☐ 5

1. **Knowledge of brain tumours influences my interest in neurosurgery or neurology as a career**

- ☐ 1 ☐ 2 ☐ 3 ☐ 4 ☐ 5

1. **I would like more teaching on neuro-oncology during medical school**

- ☐ 1 ☐ 2 ☐ 3 ☐ 4 ☐ 5

### **Section E: Open-Ended Question**

1. **In your opinion, how can medical education be improved to enhance students’ understanding of brain tumours such as meningiomas?***(Maximum 50 words)*
